# Indigenous compared with non-Indigenous Australian patients at entry to specialist palliative care: Cross-sectional findings from a multi-jurisdictional dataset

**DOI:** 10.1371/journal.pone.0215403

**Published:** 2019-05-02

**Authors:** John A. Woods, Jade C. Newton, Sandra C. Thompson, Eva Malacova, Hanh T. Ngo, Judith M. Katzenellenbogen, Kevin Murray, Shaouli Shahid, Claire E. Johnson

**Affiliations:** 1 Western Australian Centre for Rural Health, School of Population and Global Health, The University of Western Australia, Nedlands, Western Australia, Australia; 2 Cancer and Palliative Care Research and Evaluation Unit, Medical School, The University of Western Australia, Nedlands, Western Australia, Australia; 3 School of Nursing, Midwifery and Paramedicine, Curtin University, Bentley, Western Australia, Australia; 4 School of Public Health, Curtin University, Bentley, Western Australia, Australia; 5 School of Population and Global Health, The University of Western Australia, Nedlands, Western Australia, Australia; 6 Rural Clinical School, The University of Western Australia, Nedlands, Western Australia, Australia; 7 Discipline of Emergency Medicine, Medical School, The University of Western Australia. Nedlands, Western Australia, Australia; 8 Telethon Kids Institute, The University of Western Australia, Nedlands, Western Australia, Australia; 9 Centre for Aboriginal Studies, Curtin University, Bentley, Western Australia, Australia; 10 Nursing and Midwifery, Monash University, Clayton, Victoria, Australia; 11 Eastern Health, Box Hill, Victoria, Australia; University of Auckland, NEW ZEALAND

## Abstract

**Background:**

There are few quantitative studies on palliative care provision to Indigenous Australians, a population known to experience distinctive barriers to quality healthcare and to have poorer health outcomes than other Australians.

**Objectives:**

To investigate equity of specialist palliative care service provision through characterising and comparing Indigenous and non-Indigenous patients at entry to care.

**Methods:**

Using data (01/01/2010–30/06/2015) from all services participating in the multi-jurisdictional Palliative Care Outcomes Collaboration, Indigenous and non-Indigenous patients entering palliative care were compared on proportions vis-à-vis those expected from national statutory datasets, demographic characteristics, and entry-to-care status across fourteen ‘problem’ domains (e.g., pain, functional impairment) after matching by age, sex, and specific diagnosis.

**Results:**

Of 140,267 patients, 1,465 (1.0%, much lower than expected from statutory data) were Indigenous, 133,987 (95.5%) non-Indigenous, and 4,905 (3.5%) had a missing identifier. The proportion of patients with a missing identifier diminished markedly over the study period, without a corresponding increase in the proportion identified as Indigenous. Indigenous compared with non-Indigenous patients were younger (mean 62.8 versus 73.0 years, p<0.001), a higher proportion were female (51.5% versus 46.3%; p<0.001) or resided outside major cities (44.2% versus 21.5%, p<0.001). Across all domains, Indigenous compared with matched non-Indigenous patients had lower or equal risk of status requiring prompt intervention.

**Conclusions:**

Indigenous patients (especially those residing outside major cities) are substantially under-represented in care by services participating in the nationwide specialist palliative care Collaboration, likely reflecting widespread access barriers. However, the similarity of status indicators among Indigenous and non-Indigenous patients at entry to care suggests that Indigenous patients who are able to access these services do not disproportionately experience clinically important impediments to care initiation.

## Introduction

Aboriginal and Torres Strait Islander (hereafter respectfully referred to as Indigenous) people experience substantially poorer health outcomes than other Australians, with a life-expectancy gap of about a decade [1]. A considerable proportion of the gap in life expectancy is accounted for by chronic life-limiting illnesses [2]. Although the incidence rate of malignancies overall is similar among Indigenous compared with other Australians, cancers among Indigenous people tend to be diagnosed at a later stage (particularly among those residing in rural and remote areas), are more likely to be those with an inherently poor prognosis, and result in poorer survival even after stratification by stage at presentation [3, 4]. The Indigenous Australian population also has higher rates of common non-neoplastic life-limiting disorders such as chronic kidney disease [5], heart failure [6], and dementia [7]. Moreover, Indigenous patients are more likely than their non-Indigenous counterparts to encounter barriers in accessing health care [8]. The specific cultural needs of Indigenous patients are often inadequately addressed by service providers [9].

Palliative care is a holistic approach to health service provision for patients with life-limiting illnesses, along with their families, addressing psycho-social and spiritual needs as well as pain and other physical problems [10]. Recently, considerable attention in qualitative research has been devoted to exploring the experiences [11, 12] and distinctive needs [13, 14] of Indigenous Australians in palliative care. However, there is a paucity of peer-reviewed quantitative investigations of patient characteristics and service quality in this context, with no nationwide or multi-jurisdictional studies.

The Palliative Care Outcomes Collaboration (PCOC), an Australian Government-funded project, was established in 2005 for the purposes of (i) embedding standardised clinical assessment tools into routine clinical practice and (ii) systematic point-of-care data collection for reporting, benchmarking and feedback to service providers [15] ‘to support care planning and drive improvements in palliative care’ [16, p6]. Since 2010, organisations accounting for more than 80% of specialist palliative care service provision nationwide have been voluntarily submitting data to PCOC on hospital- and community-based care [15].

The current study is part of a larger research project investigating the quality and equity of palliative care provided to Indigenous Australians, using the data collected routinely by services participating in PCOC. The study’s preliminary objective arose from our observation during the initial data quality examination of frequent missing values for the Indigenous identifier and other key variables. In order to investigate further the potential impact of missing values on the validity of our findings, we extended our descriptive characterisation of patients to include individuals with a missing Indigenous identifier as a distinct group, and also examined patterns of critical missing values among patient groups and across the study period. The second objective was to characterise and thereby gauge representation of Indigenous Australians in the dataset, expanding upon the descriptive data provided in regular public-domain PCOC reporting [17]. To this end, we compared Indigenous with non-Indigenous patients in relation to their demographic, diagnostic, residential and care setting characteristics. The third objective was driven by the hypothesis that access barriers to timely care may be reflected in Indigenous patients manifesting poorer status than non-Indigenous patients at commencement of care. Accordingly, we compared the clinical and functional status of patients in these two groups at the point of entry.

## Methods

### Dataset

The hierarchically nested PCOC data comprise (i) personal details captured at entry to care by a service, (ii) information pertaining to each episode of care the patient receives, and (iii) data recorded at the beginning and end of one or more clinical ‘phases’ within each episode [16]. The dataset for this study comprised the PCOC patient records from all completed episodes of care provided by services participating in PCOC during the period 01/01/2010 to 30/06/2015. Patients are distinguishable by a numeric code, which is assigned for care within a service but does not allow between-service tracking of patients cared for by more than one participating service. The dataset did not include service identifiers or codes that would enable the researchers to distinguish individual services from one another.

Data quality was investigated in relation to missing, implausible or inconsistent data values. Discrepant data values that could not be resolved were recoded as missing. After data cleaning, the study was restricted to the initial record of patients whose first recorded episode of care by a participating service commenced on or after 01/01/2010. Lookback to the inception of PCOC in 2005 was undertaken by the PCOC data manager in order to identify and exclude from consideration the small proportion of patients in the dataset who had entered care prior to 01/01/2010 and therefore had no record of their initial episode of care in the available dataset.

### Indigenous identification and missing values

Patients were categorised as (i) Indigenous (if identified as ‘Aboriginal’, ‘Torres Strait Islander’ or ‘Aboriginal and Torres Strait Islander’); (ii) non-Indigenous (neither Aboriginal nor Torres Strait Islander) or (iii) Missing Identifier (if no Indigenous identifier was recorded). Frequencies of missing values in a range of selected variables for each group were compared. Trends in Indigenous identification over time in the proportions of Indigenous, non-Indigenous and patients with a missing identifier who had a first episode of care recorded during each month of the study period were investigated by means of a scatterplot with locally weighted smoothing.

### Patient demographic, residential, diagnostic and care setting characteristics

Patients’ demographic characteristics at entry (age, sex, and country of birth), residence at entry (jurisdiction, remoteness, and area-based socio-economic disadvantage category), primary diagnosis, and the setting of care were compared between the three groups of patients. Jurisdiction of residence categories were the six Australian states, the Northern Territory (NT), the Australian Capital Territory (ACT), and ‘Not Australia’. During the study period, no data were provided by services from the NT (2011 population ~230,000; of whom approximately 69,000 [30%] identified as Indigenous) [18], although NT-resident patients cared for elsewhere were included in the dataset. The jurisdictional categorisation of patients residing in the ACT was categorised as ‘New South Wales’ to preserve confidentiality of the single participating service in the ACT. Remoteness of residence was categorised according to the Australian Statistical Geography Standard (ASGS) (2011) [19]. Socio-economic disadvantage was categorised according to the Socio-Economic Index for Areas–Index of Relative Socioeconomic Advantage and Disadvantage (SEIFA-IRSAD) [20], a census-derived, area-level measure of social disadvantage based on location of residence. The primary diagnosis of the life-limiting condition requiring palliative care was categorised as ‘cancer’ or ‘other’, and sub-categorised by anatomical system or pathological type [21]. The setting of care was categorised as ‘admitted overnight’, ‘outpatient/ambulatory/day admission’, or ‘community-based’ [16].

### Status at entry to care

Indigenous and non-Indigenous patients were compared in relation to their palliative care problem and functional status at the start of their first episode of care. Comparisons were based on data routinely collected by participating services at the beginning and end of each clinical ‘phase’ [16, 22]. The palliative care problem status was determined using the scores from two instruments. These were (i) the Palliative Care Problem Severity Score (PCPSS), a validated clinician-rated score comprising four domains (pain, other symptoms, psychological/spiritual, and family/carer problems) [23], and (ii) the Symptom Assessment Scale (SAS), a patient-reported Likert scale of severity (0 = absent to 10 = most severe) of distress associated with seven symptom domains (pain, breathing problems, appetite problems, nausea, bowel problems, fatigue, and insomnia) [24]. Further, to investigate the comparative likelihood of multiple symptoms being reported simultaneously, the number of SAS domains scored as ‘moderate/severe’ (i.e., 4–10) were counted and the count was collapsed into binary form for the analysis (Table 1). Similarly, data on patients’ functional status were derived from the four-item Resources Utilisation Group–Activities of Daily Living (RUG-ADL) total score [25] and the Australia-modified Karnofsky Performance Status (AKPS) scale [26]. Scores from each domain were collapsed into binary form then analysed independently. The binary cut-off points were those utilised by PCOC in analyses of continuous quality improvement (PCPSS and SAS) [16], or those corresponding with a clinical recommendation to refer for review by a multidisciplinary team (RUG-ADL and AKPS) [16], as detailed in Table 1.

**Table 1:** Tools for assessing status at entry to care by a service.

Name	Domains	Scoring System	Scoring adaptation for analysis
Resource Utilisation Groups—Activities of Daily Living (RUG-ADL)		Numeric scales (1 = most independent):	Total score collapsed into binary form:
	Bed mobility	1, 3–5^a^	4–14 = relatively independent; not requiring multidisciplinary intervention^b^
	Toileting	1, 3–5^a^	15–18 = likely carer burden /pressure sore risk multidisciplinary team referral advised^b^ (‘exigent status’)
	Transfer	1, 3–5^a^	
	Eating	1–3	
	Total (all domains combined)	4–18	
Australia-modified Karnofsky Performance Status (AKPS) Scale	Consolidated measure across dimensions of activity, work, and self-care	Ordinal scale (increments of 10):	Scale collapsed into binary form:
		10 = comatose/barely rousable	10–50 = considerable assistance required multidisciplinary team referral advised^b^ (‘exigent status’)
		100 = normal, no complaints, no evidence of disease	60–100 = requires occasional assistance at most
Palliative Care Problem Severity Score (PCPSS)	Pain	Four-category ordinal scale: (absent/mild/moderate/severe)	Each domain collapsed into binary form^b^ and analysed separately:
	Other symptoms		0–1 = absent/mild
	Psychological/spiritual problems		2–3 = moderate/severe (‘exigent status’)
	Family/carer problems		
Symptom Assessment Scale (SAS)	Pain	0–10 numerical scale:	(i) Each domain collapsed into binary form^b^ and analysed separately:
	Breathing problems	0 = absent	0–3 = absent/mild
	Appetite problems	10 = most severe	4–10 = moderate/severe (‘exigent status’)
	Nausea		(ii) Number of domains simultaneously recorded as moderate/severe (i.e., scored 4–10) added—> total collapsed into binary form:
	Bowel problems		0–2 = symptoms experienced as moderate/severe
	Insomnia		≥3 = symptoms experienced as moderate/severe (‘exigent status’)
	Fatigue		

^a^ The number ‘2’ is not an available option for the Bed mobility, Toileting, and Transfer scales.

^b^ As adopted in PCOC clinical guidelines and benchmarking [16]

### Statistical analysis

Analyses were conducted using Stata Version 13.1 [27]. Chi-square and t-tests were used to compare demographic, residential, diagnostic, and setting of care characteristics of the Indigenous and non-Indigenous patient groups.

Given the potential for confounding by age, sex and underlying disease process, and the inefficiency of multivariable adjustment for ~30 specific diagnostic categories, matching was used to compare Indigenous with non-Indigenous patients in relation to status at entry. Indigenous patients selected for matching were those whose first entry to PCOC occurred during the study period, were aged ≥18 years at entry, and who had a non-missing value for age and sex and a known primary diagnosis. Matching was conducted firstly among all patients meeting these criteria, then for the subgroups cared for in the two settings (hospital-admitted overnight or community) that accounted for the majority of care episodes. Each Indigenous patient was individually matched 1:N without replacement with non-Indigenous patients who were selected randomly from among those meeting the same criteria for known matching variables, using the imatch.ado Stata command [28]. Exact matching was applied for sex and primary diagnosis. For age, the closest match was accepted (allowing for a maximum of ten years difference), with between-group equivalence confirmed by *post hoc* examination of comparative age values.

For the matched data analysis, relative risks of ‘exigent’ status (i.e., requiring immediate and/or intensive intervention) at entry of Indigenous compared with non-Indigenous patients were calculated using univariate Poisson regression with robust variance structure [29].

### Ethics

The study was approved by the Western Australian Aboriginal Health Ethics Committee (reference: #616) and the University of Western Australia Human Research Ethics Committee (reference: RA/4/1/7441). The bodies that granted permission have determined that individual consent of subjects is not required—data were analyzed anonymously and only aggregated data are presented.

## Results

The dataset comprised records of 144,951 patients. Of these, 140,267 (96.8%) had a first recorded episode of care commencing during the study period and were included in the analyses.

### Indigenous identifier

**Fig 1:**
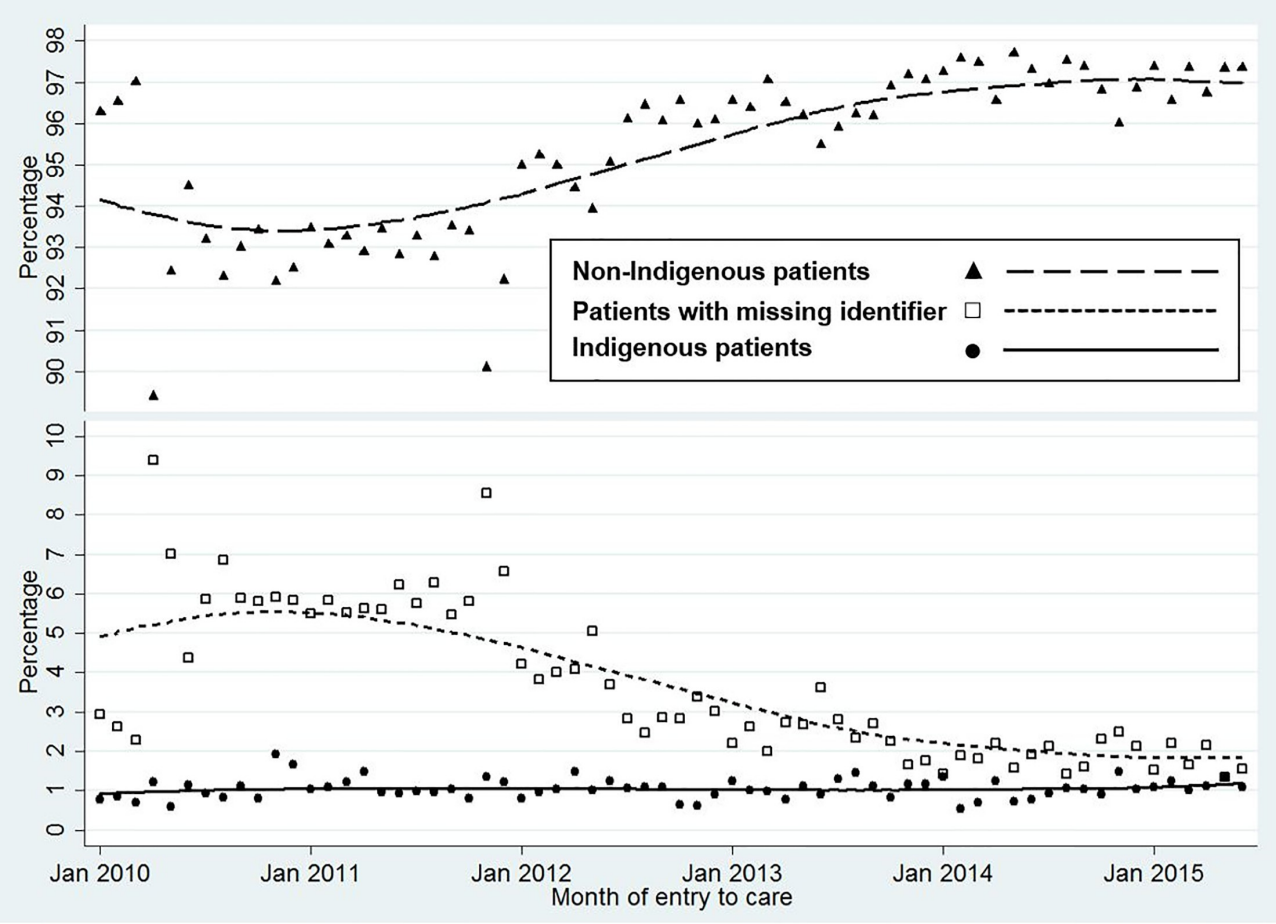
Percentage of patients in each Indigenous identifier group entering care, Jan 2010—Jun 2015. Locally weighted scatterplot smoothing curves.

Among the 140,267 patients whose first episode was captured, 1,465 (1.0%) were identified as Indigenous, 133,897 (95.5%) as non-Indigenous, and the remaining 4,905 (3.5%) had a missing identifier. The proportion of patients with a missing identifier diminished substantially over the study period, with a corresponding increase in the proportion of patients identified as non-Indigenous, while the proportion of Indigenous remained stable (Fig 1).

### Completeness of data

Patients with a missing Indigenous identifier had a much higher likelihood of missing values across other variables in the dataset than patients in either of the other two groups (Table 2). For demographic, residential and diagnostic data, at least one value of eight variables was missing in 28.2%, 26.3% and 66.3% of records (mean number of missing values 0.34, 0.32 and 1.13), respectively among patients from the Indigenous, non-Indigenous and missing identifier groups. For patient status at entry, at least one of nineteen variables was missing in 35.8%, 32.6% and 44.9% of records (mean number missing 1.10, 1.09 and 3.50), respectively (Table 2).

**Table 2:** Missing data, by patient group.

	Indigenous		Non-Indigenous		Missing identifier		Total		p-value^a^
Total patients	1,465		133,897		4,905		140,267		
**(i) Fixed patient characteristics (8 variables)**^b^									
**Zero missing values: N (%)**	1,052	(71.8)	98,677	(73.7)	1,654	(33.7)	101,383	(72.3)	
**≥1 missing values: N (%)**	413	(28.2)	35,220	(26.3)	3,251	(66.3)	38,884	(27.7)	
**Mean Number missing values**	0.34		0.32		1.13		0.35		0.21
**(ii) Patient ‘status’ at entry (19 variables)**^c^									
**Zero missing values: N (%)**	940	(64.2)	90,250	(67.4)	2,705	(55.2)	93,895	(66.9)	
**≥1 missing values: N (%)**	525	(35.8)	43,647	(32.6)	2,200	(44.9)	46,372	(33.1)	
**Mean number missing values**	1.10		1.09		3.50		1.17		0.77

^a^ Indigenous versus non-Indigenous group

^b^ Age, Sex, Specific diagnosis, Remoteness, Jurisdiction, SEIFA, Birth Country, Preferred Language

^c^ SAS pain, SAS breathing, SAS appetite, SAS nausea, SAS bowel, SAS insomnia, SAS fatigue, PCPSS pain, PCPSS other symptoms, PCPSS family problems, PCPSS psychological-spiritual, RUG-eating, RUG-mobility, RUG-transfer, RUG-toileting, AKPS, accommodation type, referral source, phase type.

Total RUG-ADL = Resources Utilisation Groups–Activities of Daily Living (total score across four domains); AKPS = Australia-modified Karnofsky Performance Status Scale; PCPSS = Palliative Care Problem Severity Score; RUG = Resources Utilisation Group; SAS = Symptom Assessment Scale.

### Patient demographic, residential diagnostic characteristics

Compared with non-Indigenous patients in the full dataset (Table 3), those identified as Indigenous were on average a decade younger (62.8 vs 73.0 years, p<0.001). A higher proportion of Indigenous patients were females (51.5% vs 46.3%, p<0.001), resided outside major cities (44.2% vs 21.5%, p<0.001) and specifically in Outer Regional, Remote or Very Remote areas (21.8% vs 5.5%, p<0.001). Also, a higher proportion resided in areas of social disadvantage (36.1% vs 21.6% in the three “Most Disadvantaged” SEIFA categories, p<0.001). Patients with a missing identifier had an average age between that of those in the two other groups (69.4 years). Statistically significant differences between all three pairs of groups were evident across most of the other characteristics investigated (Table 3). However, patients with a missing identifier were similar to the non-Indigenous group in most respects, such as the proportion of females and the proportions living outside major cities or in areas of social disadvantage.

**Table 3:** Characteristics of patients enrolled in PCOC, January 2010–June 2015 (demographic, residential, care setting and principal diagnosis category), by Indigenous identifier.

		Indigenous		Non-Indigenous		Missing Identifier		Total		p-value^a^
		**n**	**%**	**n**	**%**	**n**	**%**	**n**	**%**	
**Total: n (%)**		1465	(1.0)	133,897	(95.5)	4905	(3.5)	140,267	(100)	
**Age: mean (SD)**		62.8	(15.3)	73.0	(14.0)	69.4	(15.1)	72.7	(14.1)	<0.001
		**n**	**%**	**n**	**%**	**n**	**%**	**n**	**%**	
**Sex**	**Male**	708	(48.3)	71,800	(53.6)	2687	(54.8)	75,195	(53.6)	<0.001
	**Female**	754	(51.5)	62,036	(46.3)	2206	(45.0)	64,996	(46.3)	
	**Missing**	<5^b^	–	61	(0.1)	12	(0.2)	76	(0.1)	
Remoteness (ASGS)	Major Cities	794	(54.2)	103,207	(77.1)	3512	(71.6)	107,513	(76.7)	<0.001
	Inner Regional	327	(22.3)	21,337	(15.9)	872	(17.8)	22,536	(16.1)	
	Outer Regional	234	(16.0)	6741	(5.0)	230	(4.7)	7205	(5.1)	
	Remote	37	(2.5)	503	(0.4)	15	(0.3)	555	(0.4)	
	Very Remote	49	(3.3)	141	(0.1)	<5^b^	–	192	(0.1)	
	Missing	24	(1.6)	1968	(1.5)	274	(5.6)	2266	(1.6)	
Socioeconomic disadvantage of area (SEIFA-IRSAD) (quintiles)	1 (Most disadvantaged)	414	(28.3)	20,164	(15.1)	756	(15.4)	21,334	(15.2)	<0.001
	**2**	303	(20.7)	19,025	(14.2)	619	(12.6)	19,947	(14.2)	
	**3**	317	(21.6)	25,573	(19.1)	931	(19.0)	26,821	(19.1)	
	**4**	246	(16.8)	28,132	(21.0)	900	(18.4)	29,278	(20.9)	
	**5 (Least disadvantaged)**	161	(11.0)	38,955	(29.1)	1423	(29.0)	40,539	(28.9)	
	**Missing**	24	(1.6)	2048	(1.5)	276	(5.6)	2348	(1.7)	
**Setting of care**	**Hospital inpatient**	923	(63.0)	79,628	(59.5)	2970	(60.6)	83,521	(59.5)	<0.001
	**Hospital OP/day**	35	(2.4)	1924	(1.4)	532	(10.9)	2491	(1.8)	
	**Community**	507	(34.6)	52,345	(39.1)	1403	(28.6)	54,255	(38.7)	
	**Missing**	<5^b^	–	<5^b^	–	<5^b^	–	<5^b^	–	
**Accommodation at start of episode**	**Priv. residence**	1182	(80.7)	111,858	(83.5)	4168	(85.0)	117,208	(83.6)	<0.001
	**Other**	136	(9.3)	14,118	(10.5)	545	(11.1)	14,799	(10.6)	
	**Missing**	147	(10.0)	7921	(5.9)	192	(3.9)	8260	(5.9)	
**Jurisdiction**^c^	**NSW**	317	(21.6)	29,915	(22.3)	1234	(25.2)	31,466	(22.4)	<0.001
	**Vic**	197	(13.5)	36,673	(27.4)	1499	(30.6)	38,369	(27.4)	
	**Qld**	384	(26.2)	28,884	(21.6)	660	(13.5)	29,928	(21.3)	
	**SA**	90	(6.1)	10,409	(7.8)	488	(10.0)	10,987	(7.8)	
	**WA**	368	(25.1)	21,311	(15.9)	856	(17.5)	22,535	(16.1)	
	**Tas**	87	(5.9)	4846	(3.6)	94	(1.9)	5027	(3.6)	
	**NT**	<5^b^	–	8	(0.0)	<5^b^	–	14	(0.0)	
	**‘Not Australia’**	<5^b^	–	28	(0.0)	<5^b^	–	28	(0.0)	
	**Missing**	19	(1.3)	1823	(1.4)	71	(1.5)	1913	(1.4)	
**Principal diagnosis category**	**Cancer**	1135	(77.5)	105,060	(78.5)	4221	(86.1)	110,416	(78.7)	0.58
	**Other**	307	(21.0)	27,093	(20.2)	634	(12.9)	28,034	(20.0)	
	**Missing**	0	(0.0)	0	(0.0)	0	(0.0)	0	(0.0)	

^a^ Indigenous versus non-Indigenous group, excludes persons with missing value for characteristic.

^b^ Cells with fewer than five individuals displayed as ‘<5’ to protect identifiability.

^C^ Persons identified as residing in the Northern Territory (N = 14; <5 Indigenous) or ‘Not Australia’ (N = 28; <5 Indigenous) or Jurisdiction missing excluded from inference on proportions

ASGS = Remoteness category: Australian Statistical Geography Standard; NSW = New South Wales; NT = Northern Territory; OP/day = Outpatient or same day admission; Qld = Queensland; SA = South Australia; SEIFA-IRSAD = Socio-Economic Index for Areas–Index of Relative Socioeconomic Advantage and Disadvantage; Tas = Tasmania; Vic = Victoria; WA = Western Australia.

A higher proportion of Indigenous compared with non-Indigenous patients had care initiated in a hospital inpatient (63.0% vs 59.5%) or ambulatory setting (2.4% vs 1.4%), and a correspondingly lower proportion had care initiated in a community setting (34.6% vs 39.1%; p<0.001 for three-setting comparison). The lower proportion of Indigenous patients with care initiated in a community setting was more pronounced among patients aged <65 years (33.8% vs 39.9%; p = 0.002) than those aged ≥65 years (35.5% vs 38.3%; p = 0.07) (Table 3).

Similar proportions of Indigenous and non-Indigenous patients had cancer as their primary diagnosis (77.5% vs 78.5%, p = 0.58); a higher proportion of patients with cancer was evident in the group with a missing identifier (86.1%, p<0.001 for comparisons with both identified groups) (Table 3).

### Matched analysis—Patient status at entry to care

For all of the 1,271 Indigenous patients meeting the matching criteria, it was possible to match them to non-Indigenous patients 3:1 on both sex and specific diagnosis as well as on age within ten years (98% within two years). Accordingly, the two groups were essentially identical in average age, which was 63.4 years in both. Across all problem domains compared at entry to care, Indigenous patients had a lower or equal risk of exigent status compared with matched non-Indigenous patients (Table 4).

**Table 4:** Relative risk of palliative care problems at entry to care having ‘exigent’ status—Indigenous patients compared with non-Indigenous patients matched for age, sex and specific primary diagnosis.

	All Settings (1:3 matching; N = 5084)				Hospital Setting (1:2 matching; N = 2469)				Community setting (1:2 matching; N = 1245)			
	% Exigent status				% Exigent status				% Exigent status			
Domain	Indig	Non	RR	(95% CI)	Indig	Non	RR	(95% CI)	Indig	Non	RR	(95% CI)
**Total RUG-ADL ≥15**	26.9	26.6	1.01	(0.91–1.12)	38.2	40.6	0.94	(0.85–1.05)	8.0	8.2	0.98	(0.66–1.46)
**AKPS ≤50**	61.0	60.4	1.01	(0.96–1.06)	76.5	77.3	0.99	(0.95–1.04)	41.0	38.7	1.06	(0.92–1.22)
**SAS-Pain**	34.3	34.3	1.00	(0.92–1.09)	35.3	37.5	0.94	(0.85–1.05)	31.2	30.0	1.04	(0.87–1.24)
**SAS-Nausea**	13.1	13.9	0.94	(0.80–1.11)	14.3	16.2	0.88	(0.73–1.08)	10.5	12.8	0.82	(0.59–1.15)
**SAS-Breathing**	27.5	26.0	1.06	(0.96–1.17)	27.2	25.9	1.05	(0.92–1.20)	27.5	23.7	1.16	(0.95–1.41)
**SAS-Bowels**	19.3	20.0	0.97	(0.85–1.10)	20.4	23.5	0.87	(0.74–1.01)	16.9	18.6	0.91	(0.71–1.17)
**SAS-Insomnia**	22.8	23.3	0.98	(0.87–1.09)	21.7	24.8	0.88	(0.75–1.02)	24.2	22.1	1.09	(0.88–1.35)
**SAS-Appetite**	**30.2**	**33.5**	**0.90**	**(0.82–0.99)**	**28.2**	**33.7**	**0.83**	**(0.74–0.94)**	33.5	36.5	0.92	(0.78–1.08)
**SAS-Fatigue**	**52.2**	**56.0**	**0.93**	**(0.88–0.99)**	49.5	51.7	0.96	(0.89–1.03)	**57.0**	**65.6**	**0.87**	**(0.79–0.96)**
**≥3 Domains SAS Moderate-Severe**	**19.5**	**22.3**	**0.87**	**(0.77–0.99)**	**20.6**	**24.5**	**0.84**	**(0.72–0.98)**	17.1	19.1	0.9	(0.70–1.15)
**PCPSS-Pain**	28.6	29.8	0.96	(0.87–1.06)	31.4	33.1	0.95	(0.84–1.07)	22.4	26.5	0.85	(0.69–1.04)
**PCPSS-Other symptoms**	41.8	45.1	0.93	(0.86–1.00)	**44.2**	**48.8**	**0.90**	**(0.83–0.99)**	37.8	42.5	0.89	(0.77–1.03)
**PCPSS-Family problems**	**32.2**	**38.9**	**0.83**	**(0.76–0.91)**	**33.0**	**39.1**	**0.84**	**(0.75–0.95)**	**31.1**	**38.4**	**0.81**	**(0.69–0.96)**
**PCPSS-Psychological/Spiritual**	31.5	34.2	0.92	(0.84–1.01)	34.1	37.1	0.92	(0.82–1.03)	26.7	29.3	0.91	(0.75–1.10)

Total RUG-ADL = Resources Utilisation Groups–Activities of Daily Living (total score across four domains); AKPS = Australia-modified Karnofsky Performance Status Scale; SAS = Symptom Assessment Scale; PCPSS = Palliative Care Problem Severity Score

For both of the within-setting comparisons (Table 4), two non-Indigenous matches per Indigenous patient were attained. Essentially identical mean ages among Indigenous and non-Indigenous patients (both groups: 62.9 years in hospital setting; 64.5 years in community setting) were achieved for both of these closest-aged matched comparisons. As in the overall matched comparison, Indigenous patients in both settings had a lower or equal risk of unsatisfactory status compared with matched non-Indigenous patients across all domains. Moderate to severe family problems were reported less frequently among Indigenous patients, overall and in both settings.

## Discussion

In this large multi-jurisdictional Australian specialist palliative care dataset, patients identified as Indigenous were approximately ten years younger on average at entry to care compared with the non-Indigenous majority, and a higher proportion were females. Indigenous patients were more likely to reside outside major cities or in areas of socioeconomic disadvantage, and to commence care in a hospital setting. A comparable majority of Indigenous and non-Indigenous patients had a cancer as their principal diagnosis. The decade disparity in average age is in keeping with the well-recognised gap in life expectancy between the two groups [1]. Likewise, the modest relative female preponderance among Indigenous patients is consistent with their greater male-female disparity in deaths not amenable to palliative care (particularly those due to ‘external’ causes, e.g., injury) documented in national mortality data [30]. When matched by age, sex, and principal diagnosis, there was no difference between the two patient groups in the proportion assessed as functionally more dependent, and Indigenous patients had no greater likelihood than non-Indigenous patients of any symptom or other palliative care problem being assessed as being of moderate-severe intensity and therefore requiring urgent intervention.

However, patients included in the dataset were not representative of the Australian population during the study period. In particular, the 1.0% Indigenous patients constitute a far lower proportion than the 2.8% of persons identifying as Indigenous in national Census data (2.6% with the Northern Territory excluded) [31] and the proportion of total national deaths among Indigenous people (approx. 1.9% nationwide, 2.4% in jurisdictions with high quality Indigenous identification) [32]. In addition to the absence of participating services from the Northern Territory, which has by far the highest proportion of Indigenous population of any jurisdiction (>25%) [31], there is marked under-representation of Indigenous patients residing in Outer Rural, Remote and Very Remote ASGS areas. In national Deaths data, around three-quarters of deaths in the Indigenous population occur among those residing outside Major Cities, with about one-third reported among those from Remote or Very Remote areas [33], reflecting markedly higher proportions from these geographical areas than those among Indigenous patients with life-limiting illnesses in this study. In the Northern Territory, the differences between Indigenous and other palliative care patients in relation to age, sex, and rural/remote residence are even more marked than those in the remaining Australian jurisdictions investigated in the present study [34].

The proportion of patients identified as Indigenous over the study period remained essentially stable, while there was a marked progressive increase in the proportion of those identified as non-Indigenous and a corresponding decrease in the proportion of individuals with a missing identifier. This suggests that although ‘Missing Identifier’ patients are a heterogeneous group, the majority are likely to be non-Indigenous. Accordingly, the substantial under-representation of Indigenous patients cannot plausibly be dismissed as an artefact of data quality. Patients with a missing identifier tended to be those with a higher proportion of missing values for a range of other variables, suggesting that high frequencies of missing values occurred in services or settings where thoroughness of data collection was relatively poor (e.g., understaffed services with limited data entry capacity, or those that were still tailoring the administrative capture of PCOC data for reporting). A missing Indigenous identifier was especially frequent among patients cared for in hospital outpatient settings. Accurate identification of Indigenous Australian subjects is critically important to patient care, health service planning and meaningful investigation of equity in service provision. However, this identification remains problematic in all Australian health datasets, although data linkage has facilitated improvements in this regard for research purposes [35].

The similar patient status profiles of Indigenous and non-Indigenous patients provide some assurance that Indigenous patients are not entering care precipitously with uncontrolled symptoms or in advanced states of functional dependency due to delays or dysfunction in the processes of initiating care. To our knowledge, the only other data pertinent to this issue have been produced from our current research project based on PCOC dataset. PCOC has established a benchmark for timely care initiation, derived from the expectation that the interval between a patient being identified by a palliative care service provider as being ‘ready for care’ and the episode of care actually commencing be limited to no more than one day [16]. In a recently published paper addressing equity in attainment of this benchmark, using multi-jurisdictional PCOC data restricted to the period in which the ‘ready for care’ dates had become consistently recorded by participating services (July 2013–June 2015), we found that Indigenous patients were moderately more likely than non-Indigenous patients to experience a delay >1 day in commencement of a first episode of care (odds ratio 1.53 [95% confidence interval 1.14–2.06]), although not during second or subsequent care episodes (odds ratio 1.08 [95% confidence interval 0.61–1.90]) [36]. The findings of the current study suggest that this higher frequency of delay among Indigenous patients at entry to care by a service, while of concern, does not result in a measurably greater probability of their experiencing clinically important deterioration during the care initiation process.

For a number of the palliative care problem domains assessed, Indigenous patients were less likely than matched non-Indigenous patients to have ‘exigent’ status documented. Notably, Indigenous patients were less likely to report moderate-severe family/carer problems in the overall matched comparison as well as across both setting subgroups. However, it is not possible to exclude relative under-ascertainment of clinical problems among Indigenous patients, as these were assessed using instruments that, while validated for use in the general Australian population (23, 24), have not specifically been validated for Indigenous patients or their families, for whom communications with clinicians may be unsatisfactory [37]. Given the multiplicity of outcomes tested, there is a potential for ‘false discovery’ and a consequent need for cautious interpretation of results. Finally, even if the comparisons are valid for participating services, the under-representation of Indigenous patients likely reflects a referral bias favouring those with fewer access barriers. Also, it must be noted that service participation in PCOC is voluntary. Accordingly, it is conceivable that the quality of care—and in particular the cultural safety for Indigenous patients—provided by participating services is not indicative of specialist palliative care organisations nationwide.

The strength of this study is its foundation on a large dataset comprising records from services across multiple jurisdictions, enriched with details of patient characteristics and status. However, the interpretability of its findings is limited by under-representation of Indigenous patients (in particular, those residing outside urban areas) and by missing data. It was not feasible to address missing values with multiple imputation, given that many of the variables critical to the analysis and/or demonstrably predictive of missing values were multi-category nominal variables from which satisfactory model building is impracticable. Marked diminution in missing values during the relatively short period of the study reflects considerable efforts expended by PCOC and service providers collecting the primary information in progressively improving the quality of data collection. That this decline was unaccompanied by a rising proportion of patients identified as Indigenous suggests that those with a missing identifier did not predominantly represent a pool of Indigenous patients with disparate characteristics that could have substantially biased the representativeness of identifiable Indigenous patients.

Services participating in PCOC are named in regular public domain reporting [17], but the absence of service identifiers within the dataset precluded investigation of care according to the characteristics of individual services, such as the proportion of Indigenous patients cared for by each. Moreover, the inability to track patients cared for by more than one service will inevitably have resulted in the double-counting of some patients. In this regard, predicted higher mobility among Indigenous patients [38]—and the consequent greater likelihood of care by multiple different services during the course of a life-limiting illness—may plausibly have resulted in the disproportionate over-counting of Indigenous patients, with relative overestimation of their numbers in the dataset. Accordingly, the under-representation of Indigenous patients in care by participating services may have been even more pronounced than is evident from our analysis.

The diagnostic data provided did not include specifics of disease staging or aggressiveness. However, Indigenous patients with life-limiting illness tend to be diagnosed later, to have disease with inherently poorer prognosis (particularly in the case of common cancers [4]), and to have excess comorbidities [39]. Consequently, residual confounding by primary diagnosis after matching would be expected to accentuate problem severity and poor functional condition among Indigenous patients, and therefore this confounding is unlikely to account for their status being no worse than that of non-Indigenous patients across all problem domains. The same applies to potential confounding by remoteness of residence, for which matching was found to be impracticable.

### Conclusions

While there are caveats on the quality of data and the representativeness of participating services, the findings of this study suggest—albeit indirectly—that Indigenous patients referred to the substantial majority of services nationwide captured through participation in PCOC do not disproportionately experience problems in referral to and/or initiation of specialist palliative care services that result in their being in a poorer condition at the point of entry to care. These findings help both to map and to highlight ongoing gaps in knowledge of specialist palliative care service performance for Indigenous patients. Our ongoing research project complements this cross-sectional study with longitudinal investigation of care equity vis-à-vis the Collaboration’s quality benchmarks. Knowledge could be considerably enhanced by linkage of data, both across services participating in PCOC (a feature that is currently being established by the Collaboration), and between PCOC data and those of other core health datasets such as hospital separations, deaths, and disease registries. This would potentially facilitate detailed longitudinal investigation of care provided to patients with life-limiting illnesses throughout the entirety of their illness journeys, and could also serve to augment data quality including accuracy of identification of Indigenous patients.

## Supporting information

S1 File PCOC data access Application form (sample). (PDF)
