# Supplementary material for: Indigenous compared with non-Indigenous Australian patients at entry to specialist palliative care: Cross-sectional findings from a multi-jurisdictional dataset
Source: PLoS One. 2019 May 2;14(5):e0215403. doi: 10.1371/journal.pone.0215403 (PMC6497232; doi:10.1371/journal.pone.0215403)
Supplement: S1 File — (PDF) [file pone.0215403.s001.pdf]

Palliative Care Outcomes Collaboration

Data Access Application

Please complete this form to request information and data from the PCOC data collection.

Guidelines for application:

- Prior to completing and submitting the application, ensure that you have read the following documents, which are both available on the PCOC website, at www.ahsri.uow.edu.au/PCOC
  - *PCOC Data Policy* for information regarding use of and access to the PCOC dataset, custodianship and co-authoring arrangements
  - *PCOC Data Dictionary and Technical Guidelines f*or a description of individual data items
- Determine whether you require unit record data for analysis or aggregated (summary) data
- After completing this application form please email a copy to pcoc@uow.edu.au
- Receipt of your application will be acknowledged within 10 working days
- You may be contacted by PCOC staff for clarification of your request
- If approved, data will be released following receipt of a signed User Agreement

To request aggregated data:

- Complete Sections A, B and C
- Requests for aggregated data will be reviewed initially by PCOC staff and forwarded to the PCOC Executive Directors Group for further consideration

To request unit record data:

- Complete Sections A, B, D and E
- Requests for unit record data will be reviewed initially by PCOC staff and forwarded to the PCOC Executive Directors Group for further consideration

EXAMPLE

Office use only

Application reference:

Date received:

Action required:

Section A – Project title and applicant details

1. Project Title

2. Applicants

Include details for all project personnel, including their role in the project. Attach additional pages if necessary.

**Chief Investigator**				
Name & title:				
Organisation:				
Position:				
Role in project:				
**Project Contact**				
Name & title:				
Organisation:				
Position:				
Role in project:				
Phone:	Work:		Mobile:	
Email:				
**Co-Investigators**				
Name & title:				
Organisation:				
Position:				
Role:				
Name & title:				
Organisation:				
Position:				
Role:				
Name & title:				
Organisation:				
Position:				
Role:				
Name & title:				
Organisation:				
Position:				
Role:				
Name & title:				
Organisation:				
Position:				
Role:				

EXAMPLE

Section B – Project details

5. Does your data access application require complete project details?

Answer Yes only if your project is requires access to unit record data and/or if the PCOC team has advised you to provide full project details

☐ Yes - *please complete all questions in Section B*

☐ No – *you only require aggregate data and have been advised that it is not necessary to provide full project details. You do not need to answer any more questions in this Section. Please complete Section C.*

6. Project Description

Please provide a concise and simple description of the project in not more than 400 words

Background

Aims

Research Design

Methods

EXAMPLE

7. Duration of project

Provide the start and finish dates for the study, including data analysis

Anticipated start date:

Anticipated finish date:

8. Cohort Description

Please describe your cohort, specifying any exclusion/inclusion criteria (e.g. “all patients in the PCOC data collection” or “patients aged over 50 years referred to a specialist palliative care service in 2015”)

9. Dissemination plan

Detail how you intend to report the results of the study (e.g. publications, reports, presentations)

10. Ethics approval

Has ethical approval for this project been obtained?

☐ Yes - *please attach the application and approval letter.*

☐ No - *please explain below why ethical approval is not required.*

EXAMPLE

Section C – Application for aggregated data

11. Data description

Please provide draft tables of the information you require, including headings, selection criteria and statistical analyses (see example below). Attach additional pages as required.

Example table:

Referrals by age

Age group	*2014*		*2015*	
	Female	*Male*	*Female*	*Male*
18-25	No. (%)	No. (%)	No. (%)	No. (%)
26-35	No. (%)	No. (%)	No. (%)	No. (%)
36-45 etc…	No. (%)	No. (%)	No. (%)	No. (%)

EXAMPLE

12. Intended use of the information

If you did not provide full project details in Section B, please provide details here of how this information will be used and where it will be reported

Section D – Application for unit record data

13. PCOC data items: Please indicate the variables required for your study and provide relevant selection criteria where necessary. Refer to the PCOC Data Dictionary and Technical Guidelines for further information.

Variable	Tick if required	Selection criteria
**Patient level data items**		
Sex	☐	
State/Territory	☐	
Indigenous Status	☐	
Preferred Language	☐	
Country of Birth	☐	
Diagnosis	☐	
**Episode level data items**		
Referral Date	☐	
Referral Source	☐	
First Contact Date	☐	
Date Ready for Care	☐	
Episode Start Date (Year or Month)	☐	
Episode Type	☐	
Episode Start Mode	☐	
Accommodation at Episode Start	☐	
Episode End Date (Year or Month)	☐	
Episode End Mode	☐	
Accommodation at Episode End	☐	
Place of Death	☐	
Age group (at beginning of episode)*	☐	
Length of episode*	☐	
**Phase level data items**		
Phase Start Date	☐	
Phase Type	☐	
Phase End Date	☐	
Phase End Reason	☐	
Length of phase*	☐	
**Clinical assessments (reported at start of episode, at change in phase and at discharge)**		
RUG-ADL: Mobility	☐	
RUG-ADL: Toileting	☐	
RUG-ADL: Transfer	☐	
RUG-ADL: Eating	☐	
RUG-ADL: Total*	☐	

EXAMPLE

SAS: Insomnia	☐
SAS: Appetite Problems	☐
SAS: Nausea	☐
SAS: Bowels	☐
SAS: Breathing	☐
SAS: Fatigue	☐
SAS: Pain	☐
PCPSS: Pain	☐
PCPSS: Other Symptoms	☐
PCPSS: Psychological /Spiritual	☐
PCPSS: Family/Carer	☐
AKPS	☐

Section E - Storage and retention of data

14. Location
List all locations where the data will be stored and analysed

15. Storage of data
Please describe how the data will be stored during and after the research project

16. Security Plan
Specify the measures taken to ensure the security of information from misuse, loss or unauthorised access

EXAMPLE

17. Retention and disposal plan

Specify the period of retention of the data after completion of the project and how the information will be destroyed

EXAMPLE
